# Hypervascularized Large Vestibular Schwannomas: Single-Center Experience in a Series of Forty Cases

**DOI:** 10.1016/j.wnsx.2022.100142

**Published:** 2022-10-04

**Authors:** Luciano Mastronardi, Alberto Campione, Fabio Boccacci, Carlo Giacobbo Scavo, Ettore Carpineta, Guglielmo Cacciotti, Raffaele Roperto, Giovanni Stati, James K. Liu

**Affiliations:** 1 Department of Neurosurgery, San Filippo Neri Hospital/ASL Roma 1, Rome, Italy; 2 Department of Neurological Surgery, Rutgers University-New Jersey Medical School, Saint Barnabas Medical Center, RWJ Barnabas Health, Livingston and Newark, New Jersey, USA

**Keywords:** Adhesions, Facial nerve, Hypervascular, Microneurosurgery, Vestibular schwannoma, ASA, American Society of Anesthesiologists, FN, Facial nerve, HB, House-Brackmann, HVVS, Hypervascular vestibular schwannoma, MRI, Magnetic resonance imaging, NS, Not significant, SRS, Stereotactic radiosurgery, VB, Vertebral-basilar, VS, Vestibular schwannoma

## Abstract

**Background:**

Vestibular schwannomas (VS) are usually hypovascularized benign tumors. Large VS (Koos grade IV) with unusual vascular architecture are defined as hypervascular (HVVS); the excessive bleeding during microsurgery has a negative impact on results.

**Methods:**

Forty consecutive patients were operated on for HVVS (group A). A tendency to bleed and adherence of capsule to nervous structures were evaluated by reviewing intraoperative video records. The cisternal facial nerve (FN) position was reported. Microsurgical removal was classified as total, near-total, subtotal, or partial and the MIB-1 index was evaluated in all. FN results were classified according to the House-Brackmann scale.

**Results:**

Results of Group A were compared with those of 45 patients operated on for large low-bleeding VS (group B). Mean tumor diameter was 3.81 cm in group A and 3.58 cm in group B; the mean age was 42.4 and 56.3 years, respectively. The mean American Society of Anesthesiologists Physical Status Scale class of group A was 1.67 versus 2.31 of group B (P < 0.01). Total or near-total resection was accomplished in 76.5% of group A versus 73.3% of group B. Tight capsule adhesion was observed in 67.5% of group A versus 57.8% of group B. Mean MIB-1 was 1.25% and 1.08%, respectively.

FN anatomic preservation was possible in 84.6% of group A versus 95.5% of group B; 67.5% of group A had HB grade I or II FN outcome versus 93.3% of group B (*P* < 0.001). In group A, 8 patients (20.0%) experienced transient postoperative complications versus 4.4% of group B. Recurrence/regrowth was observed in 4 patients in group A versus 1 in group B.

**Conclusions:**

Intraoperative video for classification of HVVS was used. Microsurgery of large HVVS was associated with higher (usually transient) complications and recurrence/regrowth rates and poorer FN outcome, especially in patients with tight capsule adhesion.

## Introduction

According to the Koos classification,^1^ grade IV vestibular schwannomas (VS) are large tumors compressing the brainstem, displacing the fourth ventricle. During recent decades, their incidence has reduced as a result of broad access to magnetic resonance imaging (MRI).^2^, ^3^, ^4^ In the past, they represented 40% of all VS, whereas they have accounted for only a few percent in the last 10 years.^5^ Surgical resection is the treatment of choice.^5^, ^6^, ^7^

Microsurgery of large VS is challenging because of common adhesions between the tumor capsule and the brainstem and the facial nerve (FN) and, in several cases, tendency of the tumor to bleeding. In particular, even if most VS are hypovascular tumors, some tumors have an unusual vascular architecture and are defined as hypervascularized VS (HVVS). The rate of HVVS increases with size^8^ and its incidence seems to be higher in large and solid VS and in young people.^9^ The blood supply of VS comes from branches of the external carotid artery and/or from the vertebral-basilar (VB) system.^8^^,^^9^ According to the angiographic study of Teranishi et al.,^8^ HVVS have a high concentration of abnormal vessels, with or without arteriovenous shunts.

Few investigators^8^, ^9^, ^10^ have analyzed in detail the outcome of HVVS compared with hypovascular VS. By using a novel application consisting of reviewing intraoperative video records to evaluate the tendency to bleed and adherence of capsule to nervous structures, we retrospectively analyzed the clinical and surgical data and the outcome of a series of 40 patients with Koos grade IV HVVS^1^ consecutively operated on by retrosigmoid approach. The extent of tumor removal, FN outcome, and complications were highlighted, compared with 45 low-bleeding VS, operated on during the same period.

## Methods

### Study Design

This single-center study was carried out at a tertiary-care referral hospital. It was conducted after obtaining clearance from the internal ethics committee of our institution and in accordance with the principles of the Helsinki Declaration. Written consent for scientific treatment of personal data was obtained from each patient before surgery. Cohorts included all patients who underwent surgery in the San Filippo Neri Hospital of Rome for Koos grade IV VS between December 2010 and September 2021: 40 with HVVS (group A) and 45 with low-bleeding VS (group B), of 285 patients with unilateral VS surgically treated by the first author in the same period. All patients were followed up until March 2022.

### Data Collection

Data were collected from case sheets, operative notes, neuroimaging PACS (picture archiving and communication system), and discharge summaries. Subsequently, the patients were followed up on an outpatient basis. The parameters studied included demographic profiles, clinical features, duration of symptoms, neurologic status, tumor size, neuroimaging, operative details, histopathologic data, recurrence, functional outcome, mortality, and morbidity.

**Figure 1:**
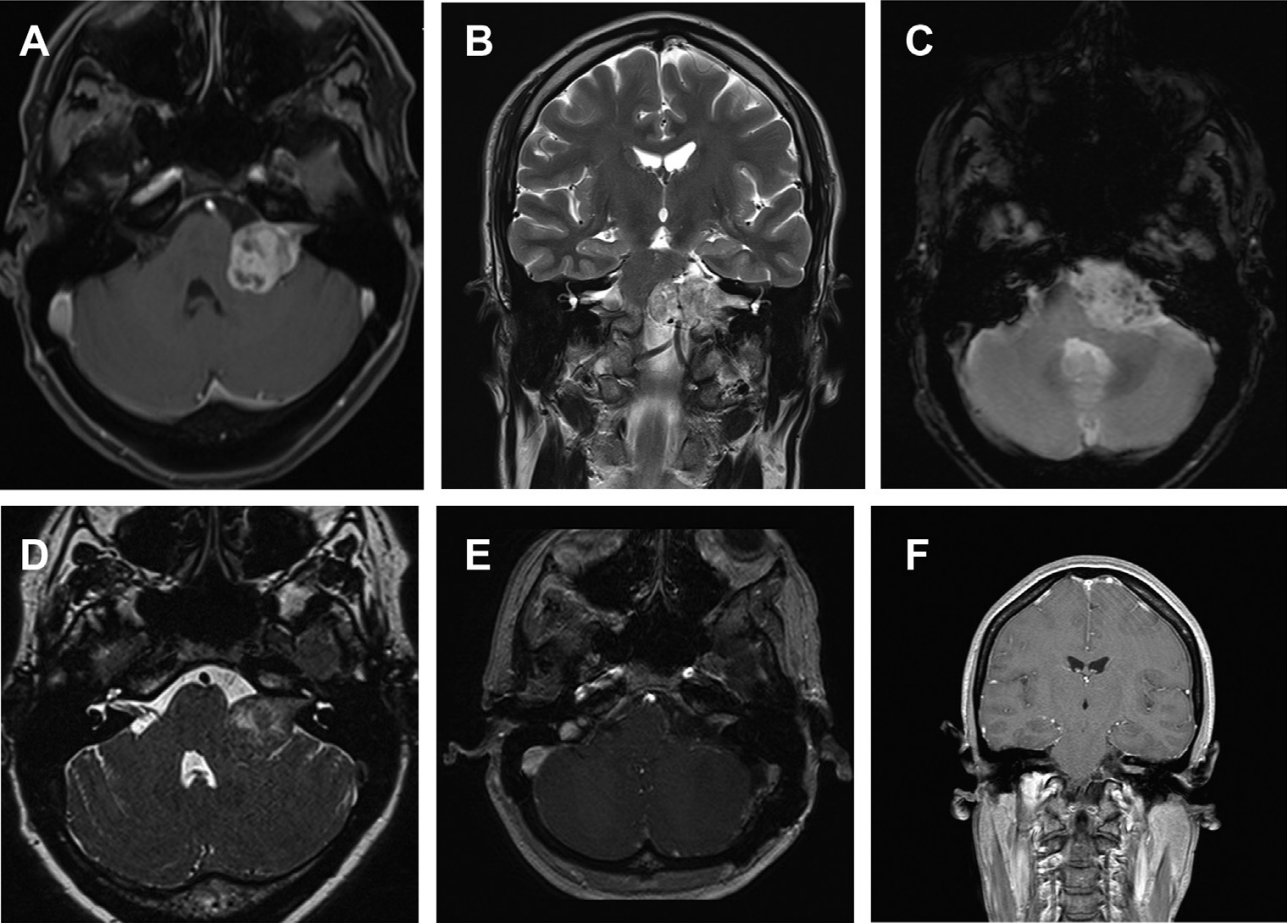
Preoperative and postoperative magnetic resonance imaging (MRI) of a 43-year-old woman with left Koos grade IV hypervascular vestibular schwannoma: near-total resection, without any neurologic deficit (House-Brackmann grade I facial nerve result), except left hearing loss (preoperative American Academy of Otolaryngology–Head and Neck Surgery class C). (**A**) Axial slice of preoperative MRI-T1 gadolinium (Gd) showing a Koos grade IV left vestibular schwannoma. (**B**) Coronal slice of T2 MRI, showing several serpiginous vessels inside the mass. (**C**) Axial slice of T2 MRI showing multiple black spots, representing sections of intratumoral vessels. (**D**) Axial slice of three-dimensional T2 MRI, showing an inhomogeneous tumor, with multiple spots inside, especially close to the interface with brainstem. (**E**) Axial slice of postoperative T1 MRI Gd showing minimal residual tumor in the internal auditory canal. (**F**) Coronal slice of postoperative T1 MRI Gd.

General conditions and preoperative risk were assessed according to the American Society of Anesthesiologists (ASA) classification.^11^ Patients with neurofibromatosis type 2 were not included. Preoperative neuroimaging included temporal bone computed tomography and contrast-enhanced MRI in all. Tumor size was categorized according to the international criteria, measuring the largest tumor diameter on postcontrast axial MRI.^12^ The MRI slices of HVVS consistently showed multiple serpiginous flow voids, representing large feeders and draining veins within the mass, especially in T2-weighted images (Figure 1). Even if the definition of HVVS is based on the abnormal vascular architecture of tumor in the angiographic study,^8^ we seldom perform this examination in the preoperative setting of posterior fossa tumors, because of possible risks related to the procedure.

Preoperative audiovestibular evaluation included pure tone audiometry and speech audiometry. Hearing level was assessed according to the American Academy of Otolaryngology–Head and Neck Surgery classification^13^: class A and B were categorized as good hearing.

Microsurgery via retrosigmoid approach was performed in all. Location of FN and its adherence to the tumor were evaluated by reviewing radiologic images and surgical and video records. The course of FN was classified according to its position in relation to the tumor: anterior, anterior-inferior, anterior-superior, and dorsal^14^^,^^15^; anterior and inferior and anterior-superior positions were classified as polar, whereas anterior and dorsal were classified as equatorial in relation to the mass. At the end of microsurgical resection, a flexible video endoscope (4 mm × 65 cm; Karl Storz, Tuttlingen, Germany) was inserted in the surgical cavity, handled by the surgeon,^16^^,^^17^ to detect possible tumor residues in the internal auditory canal. The extent of tumor removal was classified as total, near-total (tumor residue <5%), subtotal (residue 5%–10%), and partial (residue >10%). Extent of resection was assessed by blind review of postoperative MRI performed by 1 coauthor and 1 neuroradiologist.

On pathologic examination, the MIB-1 index was evaluated in all cases.

FN function, classified according to the House-Brackmann^18^ (HB) grading system, was evaluated preoperatively, at discharge, and at final follow-up (≥6 months).

### Neurophysiologic Monitoring

The surgical procedures were performed with intraoperative FN monitoring (Nimbus i-Care 100 [Newmedic Division, Hemodia, Labège, France]), with electrodes inserted in orbicularis oris and orbicularis oculi muscles. Nerve stimulation was performed with monopolar probes to locate the FN and verify its function.

### Operative Technique

In all cases, the lateral position was adopted. A particular attention was always paid to correct neck flexion, to avoid possible jugular vein compression.

The retrosigmoid approach was used in all patients. A slightly curved 5-cm to 6-cm skin incision was performed behind the ear, 1–2 cm posteriorly to the mastoid process. The lateral occipital bone was exposed, including superior and inferior nuchal lines. About 3 cm^2^ retrosigmoid suboccipital craniotomy was realized, exposing the sigmoid and transverse superiorly. The dura was incised in a semicircular fashion, the lateral medullary cistern was opened, and the cerebrospinal fluid aspirated to obtain cerebellar decompression.

We always avoid coagulating the superior petrosal veins and other large veins.

After cutting the dura along the Tübingen line^19^ (the dural landmark of the inferior limit of the internal auditory canal on the posterior surface of petrous temporal bone), the canal was opened through an extracoarse diamond burr or by ultrasonic aspirator. The tumor surface was exposed and the possible dorsal displacement of the FN was investigated by stimulation. The subperineural (subcapsular) plane^20^ was identified for debulking, detachment, and final dissection.

A V cut was performed on the dorsal surface of the tumor with microscissors or handheld thulium laser and debulking was obtained with Sonopet Ultrasonic Aspirator (Stryker, Kalamazoo, Michigan, USA), microscissors, microcurettes, low-power bipolar forceps, and laser. In particular, for capsule incision, cutting, vaporizing, coagulating, and debulking the mass, we used a handheld 2μm-thulium flexible laser fiber (Revolix Jr [Lisa Laser, Pleasanton, California, USA]). Irrigation with standard saline solution was used for cooling surrounding tissues and the fiber, which was not hindered in its function by the water.

After debulking, the remaining tumor capsule was dissected from the brainstem and cranial nerves during continuous FN monitoring, using standard microsurgical tools. When possible, we tried to follow the arachnoid reflection over the cranial nerves and brainstem to obtain the best functional results.

In some cases of strong tumor adherence to the FN and/or brainstem, a millimetric remnant of tumor capsule was left, yielding a near-total resection. In a few patients with tight tumor capsule adherences and a high bleeding rate of tumor, a subtotal removal was performed. Accurate hemostasis, mastoid cells obliteration, and tight dura closure by pericranial graft, hemostatics, and sealants were obtained; the bone operculum was placed again with miniscrews.

### Bleeding Rate and Adhesion of Tumor Capsule

To assess the tendency of tumor to bleed and the adherence of capsule to the FN and brainstem, 2 independent blinded reviewers (expert coauthors) expressed their opinion watching the surgical video, without considering the impressions of the surgeon described in the report. The novel application to use intraoperative video to evaluate the tendency to bleed and adherence of capsule to the nervous structures of large VS was preferred to the more invasive preoperative angiography previously described.^8^

Bleeding tumors, classified as HVVS, had an unusual and redundant superficial and internal vascular architecture. Except for the angiographic classification proposed by Teranishi et al.,^8^ in the literature, there is no objective definition of hypervascularity. During surgery, in all these cases, intratumoral and extratumoral vascularity was arterialized for luxurious shuntings and removal was complicated by high-rate bleeding during debulking and dissection, because of the rupture of the multiple vessels in the capsular and intracapsular portion of tumor. Possible high bleeding behavior of VS was suspected on preoperative T2-weighted MRI when multiple serpiginous flow voids, representing large feeders and draining veins, were detected within the mass (Figure 1). The blind independent observers rated the bleeding amount and classified tumors as hypervascularized and hypovascular. Agreement was reached in all cases.

MRI findings of flow voids correlated with vascularity as assessed by the observers.

VS without a clear perineural dissection plane between nervous structures (in particular, FN and brainstem) and tumor capsule were considered more adherent, compared with VS with a well-recognizable dissection plane (less adherent). There is no scale for the degree of adhesion; therefore, less strong adherences were defined as those in which the FN and brainstem could be separated from the tumor comparatively easily with a microdissector, and strong adherences were defined as those that were difficult to separate, requiring sharp dissection with microscissors. In patients in whom adhesions were too tight and in the presence of brainstem edema, total resection was considered unsafe and less than total resection was accepted.

### Clinical Follow-Up

Clinical and radiologic follow-up was scheduled at 6 months after operation and then once a year; the follow-up period ranged between 6 and 138 months (mean, 71 months; median, 73 months). FN outcome evaluation was rated at 6 months after operation and at last follow-up and was categorized according to HB grades I–VI.

### Statistical Analysis

Statistical analysis was performed by means χ^2^ test for categorical variables and Student *t* test for continuous variables, using the software MS Excel (Microsoft Corp., Redmond, Washington, USA). Statistical significance was set at *P* < 0.05.

## Results

### Characteristics of the Patients and Clinical Presentation

The maximal mean tumor diameter in group A was 3.81 cm (range, 3.1–5.5 cm) compared with 3.58 cm (range, 3.1–4.5 cm) in group B (*P* not significant [NS]). Among 40 patients in group A, 21 were male and 19 female; in group B, 19 were male and 26 female. The mean age of patients in group A was 42.4 years (range, 22–64 years; standard deviation, 13.2), versus 56.3 years (range, 23–80 years; standard deviation, 25.7) in group B (NS). The preoperative general conditions of patients with HVVS was better than those with hypovascular VS: the mean ASA class was 1.67 in group A (range, 1–2) versus 2.31 (range, 1–3) in group B (*P* < 0.01), in accordance with the younger age of group A. In 7 patients with VS in group A (17.5%) and in 13 patients in group B (28.9%) 1 or more cysts were present (NS).

Severely impaired hearing or deafness was present in 39 patients in group A (97.5%) and in 42 patients in group B (93.3%); hearing preservation was not possible in any cases because of the large size of the tumors and the consequent impossibility to safely detach the cochlear nerve from the tumor capsule.

Preoperative HB grade II–IV FN deficit was observed in 6 patients with HVVS (15.0%) and in 3 (6.7%) with low-bleeding VS (NS).

Patients’ preoperative data are summarized in Table 1.

**Table 1:** Preoperative Clinical and Radiologic Data of the Study Population

Characteristics	Group A (N = 40): Hypervascular Vestibular Schwannoma	Group B (N = 45): Low-Bleeding Vestibular Schwannoma	χ^2^ Test, *P*
Sex, n (%)			
Female	19 (47.5)	26 (57.8)	0.12
Male	21 (52.5)	19 (42.2)	
Age (years), mean ± standard deviation (range)	42.4 ± 13.2 (22–64)	56.3 ± 25.7 (23–80)	0.39
Diameter (cm), mean (range)	3.81 (3.1–5.5)	3.58 (3.1–4.5)	0.28
American Society of Anesthesiologists grade (range 1–3), mean	1.67	2.31	<0.01
≥1 cysts, n (%)	7 (17.5)	13 (28.9)	0.09
Hearing loss (American Academy of Otolaryngology–Head and Neck Surgery grade), n (%)			
C	18 (45.0)	20 (44.4)	NS
D	21 (52.5)	22 (48.9)	
Preoperative facial nerve deficit (House-Brackmann grade)			
II	3	1	NS
III	2	2	
IV	1	0	

NS, not significant.

### Extent of Resection, MIB-1 Index, and Adhesions of Tumor Capsule

Total or near-total resection was obtained in 65 patients (76.5%): 32 patients with HVVS (80.0%) and 33 (73.3%) with hypovascular tumors (NS). There were no significant differences in the residual tumor volume in the 2 groups. Mean MIB-1 index was 1.25% (range, 1–3) in group A and 1.08% (range, 1–2) in group B (NS).

Tight adhesion of capsule to nervous structures was observed in 27 patients with HVVS (67.5%) versus 26 patients with low-bleeding tumors (57.8%) (NS).

On considering both groups, time of surgery ranged from 180 to 390 minutes (mean, 303 ± 37 minutes): 327 ± 41 minutes in group A versus 283 ± 31 minutes in group B (NS). The blood loss ranged from 50 mL to 350 mL (mean, 220 ± 24 mL) and 265 ± 27 mL in group A versus 155 ±18 mL in group B (NS).

Data related to surgical and pathologic details are summarized in Table 2.

**Table 2:** Extent of Surgical Removal and Age, MIB-1 Index, Adhesion of Capsule, and Recurrence Rate

		Group A (40 Patients)	Group B (45 Patients)	χ^2^ Test, *P*
Extent of resection, n (%)				
Total/near-total		32 (76.5)	33 (73.3)	0.39
Subtotal		8 (23.5)	12 (26.7)	
MIB-1 index (%), mean (range)		1.25 (1–3)	1.08 (1–2)	0.71 (*t* test)
Capsule adhesion to nervous structures, n (%)				
Yes		27 (67.5)	26 (57.8)	0.43
No		13 (32.5)	19 (42.2)	
Recurrence rate, n (%)	5 (5.9)	4 (10.0)	1 (2.2)	0.002

### Long-Term FN Outcome

Anatomic preservation of FN was possible in 33 HVVS (84.6%) and in 43 low-bleeding tumors (95.5%) (*P* < 0.001). An anterior-superior position of the FN was observed in 47.5% of patients in group A (*n* = 19) and in 24.4% of patients in group B (11 patients) (*P* < 0.001).

Twenty-seven patients in group A (67.5%) had a long-term HB grade I–II FN result versus 42 in group B (93.3%) (*P* < 0.001). Capsule of tumor strongly adherent to nervous structures was observed in 27 patients in group A (67.5%) and in 26 patients in group B (57.8%) (NS). Data related to FN outcome are summarized in Table 3.

**Table 3:** Facial Nerve Position and Results

	Group A (N = 40), n (%)	Group B (N = 45), n (%)	χ^2^ Test, *P*
Anatomic preservation of facial nerve			
Yes	33 (84.6)	43 (95.5)	<0.001
No	7 (15.4)	2 (4.5)	
FN position in the cisternal segment			
Anterior-superior	19 (47.5)	11 (24.4)	<0.001
Anterior/anterior and inferior	21 (52.5)	34 (75.6)	
FN results at follow-up (House-Brackmann grade)			
I–II	27 (67.5)	42 (93.3)	<0.001
>III	13 (32.5)	3 (6.7)	

FN, facial nerve.

### Postoperative Complications and Recurrences

Mortality was zero in both groups.

Transient postoperative complications were observed in 8 patients in group A (20.0%) versus 2 patients in group B (4.4%) (*P* < 0.05). In particular, wound infection (3 patients), transient diplopia (2 patients), cerebellar mutism, dysphagia, pneumonia (resolved with antibiotics), cerebellar infarction, and hydrocephalus (resolved with ventricular-peritoneal shunt) in 1 each. One patient with HVVS had permanent diplopia for abducens nerve paralysis.

At a mean follow-up of 71 months (median, 73 months), a recurrence/regrowth of tumor was observed in 4 patients operated on with ST removal of HVVS (2 surgery, 2 stereotactic radiosurgery [SRS]) and in 1 patient in group B, who preferred to be reoperated on (NS). On the whole, reoperation was considered in all patients with progressive regrowth of VS; SRS was preferred in older patients, in those with ASA class >III general conditions and in those who refused second surgery.

Complications and recurrences are summarized in Table 4

**Table 4:** Mortality and Morbidity (Permanent and Transient Complications)

Mortality, Morbidity, and Second Surgery for Recurrence	Group A (N = 40)	Group B (N = 45)	χ^2^ Test, *P*
Mortality (n)	0	0	
Transient complications, n (%)	8 (20.0)	2 (4.4)	<0.05
Permanent complications (n)	1 (diplopia for abducens nerve paralysis)	0	
Recurrences (n)	4 (2 surgery and 2 stereotactic radiosurgery)	1 (surgery)	Not significant

## Discussion

Large VS are challenging tumors, especially in patients with adherences of capsule to nervous structures (i.e., to the brainstem and FN) and with a high concentration of abnormal vessels and arteriovenous shunts.^8^ Few studies^8^, ^9^, ^10^ have reported clinical results of HVVS compared with low-bleeding VS. The main limitations of this study are the lack of a proper definition of HVVS, the subjective blinded allocation of the VS to the hypervascularized or low-bleeding group, and its retrospective nature.

According to Yamakami et al.,^9^ HVVS are large and solid tumors, presenting often in younger patients. Also in our series, hypervascularized tumors were usually solid Koos grade IV VS and the mean age was lower than that of patients with low-bleeding VS. In addition, the preoperative mean ASA class of patients with HVVS was better than that of hypovascular patients: it is reasonable to believe that this difference could to be partially attributable to the younger mean age of patients in group A.

### Large and Bleeding VS

The relevance of blood supply for microsurgery and outcome of VS has been seldom reported in the literature.^8^^,^^21^^,^^22^ Although preoperative angiography provides characteristic findings,^8^ MRI is useful too, showing multiple flow voids inside the tumor (Figure 1). Teranishi et al.^8^ proposed a classification of HVVS in 5 types in relation to tumor feeders and to the presence of arteriovenous shunts. The presence of shunts was less frequent but was associated with a higher rate of recurrence, especially if feeders come VB system and external carotid artery branches and if arteriovenous shunts originated from VB arteries. In our study, we proposed the use of preoperative MRI features combined with intraoperative video for classification of large HVVS as a novel application, less invasive than the preoperative angiography previously described.^8^

Some investigators^9^^,^^10^ have proposed 2-step surgical treatment of HVVS, assuming that partial resection could reduce the vascularization of tumor, making total removal less risky in the second surgical step. We agree with Teranishi et al.^8^ to attempt total or near-total resection during the first surgery, for overall comfort of patients and for reducing the risk of postoperative hemorrhage after the first step.

According to Peris-Celda et al.,^23^ large VS are more frequent among younger patients, postulating more aggressive tumor biology. In particular, on comparing VS with diameter >4 cm with the rest of the cohort, these investigators observed a significant difference of mean age at diagnosis: 52.3 years for smaller versus 42.4 years for larger tumors.^23^

Angiogenesis is essential for the enlargement of solid tumors, including schwannomas, and vascular endothelial growth factor expression of VS is a strong mediator of tumor angiogenesis, correlating with tumor growth pattern.^24^, ^25^, ^26^, ^27^ Moller et al.^28^ observed that the concentration of metalloproteinase 9 correlates with VS growth rate and its adhesion to nerve structure. In addition, the expression of MEK/ERK effectors, oncogenic gene miR-21, and mTOR (mammalian target of rapamycin) pathways involved in several cellular processes^29^ could explain the relationships among vascularization, adherences, and size.

Few studies have attempted to profile genome-wide alterations in sporadic VS. In a series of 23 VS, Carlson et al.^30^ analyzed fresh frozen tumor specimens and matched peripheral blood leukocytes, to identify if more clinically aggressive variants possess specific genetic alterations. Using high-throughput deep sequencing, 2-hit alterations in the neurofibromatosis 2 (NF2) gene were identified in every tumor and not in peripheral blood, supporting that all events were somatic.^30^

### Surgical Dissection for Large, Vascularized, and Adherent VS

The layers encountered from the surface of VS are 1) arachnoid folder; 2) FN and cochlear nerve; and 3) perineurium/nerve fibers of vestibular nerve of origin of VS (capsule).^24^^,^^25^^,^^31^ In some Koos grade IV VS, there is no arachnoid separating the capsule from FN and cochlear nerve.^20^

The planes for possible tumor dissections are A) subarachnoid; B) subperineural (subcapsular); and C) intracapsular.^20^ During the removal of large VS, Kohno et al.^20^ suggest paying attention to tumor capsule and arachnoid reflection.^2^^,^^32^, ^33^, ^34^, ^35^ Epiarachnoid tumors do not have a second layer of arachnoid on the tumor after moving the arachnoid fold toward the brainstem. In contrast, subarachnoid VS maintain a layer of arachnoid on the capsule after moving the arachnoid fold.^33^ On light and electron microscopy, Kohno et al.^20^ confirmed the existence of an arachnoid membrane covering the capsule after the removal of the arachnoid fold in most cases (subarachnoid VS).

Notwithstanding the knowledge of these technical refinements and attentions, microsurgery of Koos grade IV HVVS is usually difficult, especially in patients in whom the capsule is adherent to the brainstem and FN, because of loss of visualization resulting from intraoperative bleeding and disruption of dissection planes.

### Extent of Removal of Large VS and Markers of Tumor Cell Proliferation

Large size often compromises safe and effective resection: the rate of total removal ranges between 28.6% and 95.5%^36^^,^^37^; in 2 series, total resection could be accomplished in all cases.^38^^,^^39^ Factors that negatively affect results, such as hypervascularity, determine intraoperative high bleeding and increased technical difficulties.^8^, ^9^, ^10^

In our series of large HVVS, total or near-total resection was accomplished in 76.5% of patients, in line with the literature.^36^^,^^40^, ^41^, ^42^ In addition, tight adhesion of capsule was observed in 67.5% of HVVS versus 57.8% of low-bleeding VS (Table 2).

To limit heat thermal damages, a 2μm-thulium handheld laser resulted to be useful and safe for cutting, vaporizing, and coagulating the capsule and the intracapsular part of tumor, in combination with low-power bipolar coagulation, microscissors, and ultrasonic aspirator.^43^

Antigen Ki-67, tested with the MIB-1 index, is a nuclear protein associated with cellular proliferation. VS with MIB-1 index higher than 3% are actively proliferating with theoretic higher risk for regrowth.^44^ According to Teranishi et al.,^8^ HVVS have a significant higher MIB-1 index (4.3% vs. 2.8% of low-bleeding tumors). These data are in contrast with our series, in which mean MIB-1 index was 1.25% in group A and 1.08% in group B.

### Functional Results

Hearing loss is one of the most common presenting signs of VS (41.7% of cases)^45^; if hearing is preoperatively socially useful, attempts should be made to accomplish its preservation, especially in small tumors.^46^ On considering large VS, in selected series, hearing has been preserved in 21.4%–66.7% of cases.^46^, ^47^, ^48^, ^49^, ^50^ In our series, preoperative severely impaired hearing or deafness was present in all patients in group A and in 92.0% of patients in group B.

Although great emphasis is placed on preserving FN function, its injury still represents a relatively common complication, especially in large tumors, and an anatomically intact nerve does not necessarily predict an HB grade I function. According to the literature, preservation of FN in VS surgery is accomplished in 32.9%–83.3% of cases.^36^, ^37^, ^38^, ^39^, ^40^^,^^50^, ^51^, ^52^ In our entire cohort, postoperative HB grade I–II outcome was observed in 81.2% of patients (69 of 85) and FN anatomic preservation was possible in 84.6% of HVVS and in 95.5% of low-bleeding tumors. HB grade I–II FN result at last follow-up was observed in 67.5% of patients in group A versus 93.3% of patients in group B (*P* < 0.001) (Table 3).

The worst FN outcome in HVVS surgery induced some surgeons to leave more residue. Zhang et al.^25^ obtained the best functional outcome in patients who underwent subtotal instead of radical resection. Even if controversial results have been reported with planned less than total resection for FN preservation, according to some investigators,^53^, ^54^, ^55^, ^56^, ^57^ outcome might be improved in selected patients treated with combined surgical/radiosurgical treatment. Zumofen et al.^57^ reported 89% HB grade I–II postoperative rate, with no need for salvage surgery after Gamma Knife surgery (Elekta, Stockholm, Sweden) on planned tumor residues. However, Iwai et al.^54^ found that optimal FN outcome (95% postoperative HB grade I–II) could be jeopardized by the need of salvage surgery after Gamma Knife in case of large residues (>6 cm^3^).

### Complications and Recurrences/Regrowth of Residue

In our series, mortality was zero and a permanent complication (diplopia for sixth nerve paralysis) occurred in only 1 HVVS. Transient postoperative complications were observed in 8 patients with HVVS (20.0%), without correlation with preoperative ASA class. At a follow-up ranging from 6 to 138 months, recurrence/regrowth of residue was observed in 5 patients: a reoperation was performed in 2 patients with HVVS and in 1 patient with low-bleeding VS, and SRS in another 2 patients with HVVS (1 patient who showed ASA class 3 and 1 patient who refused second surgery). It is unclear if the higher rate of recurrence in HVVS could be attributable to vascularity and to mediators of angiogenesis, correlated with tumor growth pattern.^24^, ^25^, ^26^, ^27^

These rates are in line with the literature^6^^,^^7^^,^^37^^,^^38^^,^^40^^,^^45^^,^^51^^,^^54^^,^^57^^,^^58^ and confirm that a retrosigmoid approach is safe even in giant VS.^6^^,^^37^^,^^38^^,^^49^, ^50^, ^51^^,^^58^ The translabyrinthine approach has been traditionally suggested for large tumors, with good results in terms of extent of resection (rates of total resection around 90%), postoperative facial outcome (HB grade I–III around 75%) and perioperative complications (cerebrospinal fluid leaks about 2%).^36^^,^^59^, ^60^, ^61^ On the other hand, other investigators reported perioperative complication rates as high as 14.3%.^62^ Even if a translabyrinthine approach is a feasible alternative, the results of our series contribute to supporting the use of the retrosigmoid approach in large HVVS.

Surgical resection represents the ideal treatment for large and giant VS, including HVVS. It significantly and positively affects the patients’ quality of life^7^ and should be considered even in elderly patients.

### Limitations of the Study

The major limitations of this study are the lack of a proper definition of vascularity of VS, the lack of a proper definition of adhesion of tumor capsule, and the retrospective nature of the study.

## Conclusions

In this study, we proposed the use of preoperative MRI features combined with intraoperative video for classification of large HVVS as a possible novel application, less invasive than the preoperative angiography previously described.^8^

Compared with low-bleeding VS, microsurgery of large HVVS seems to be associated with a higher complication rate, higher recurrence/regrowth rate, and poorer FN outcome, especially in patients with tight capsule adhesions.

## CRediT authorship contribution statement

**Luciano Mastronardi:** Methodology, Conceptualization, Formal analysis, Writing – original draft. **Alberto Campione:** Formal analysis. **Fabio Boccacci:** Formal analysis, Data curation. **Carlo Giacobbo Scavo:** Formal analysis. **Ettore Carpineta:** Formal analysis. **Guglielmo Cacciotti:** Formal analysis. **Raffaele Roperto:** Formal analysis, Writing – review & editing. **Giovanni Stati:** Writing – review & editing. **James K. Liu:** Writing – review & editing, Supervision.

## DECLARATION OF COMPETING INTEREST

Conflict of interest statement: The authors declare that the article content was composed in the absence of any commercial or financial relationships that could be construed as a potential conflict of interest.
